# Patterns of Amphotericin B Use and Factors Related to Mortality in a Low-Middle Income Country: An Observational and Longitudinal Study

**DOI:** 10.3390/antibiotics13111015

**Published:** 2024-10-29

**Authors:** Luis Fernando Valladales-Restrepo, Lian Manuela Soto-Romero, Luis Fernando Navarrete-Santa, Rodrigo Montoya-García, Jaime Andrés Ríos-Montoya, Alejandra Sabogal-Ortiz, Jorge Enrique Machado-Alba

**Affiliations:** 1 Grupo de Investigación en Farmacoepidemiología y Farmacovigilancia, Universidad Tecnológica de Pereira-Audifarma S.A., Pereira 660003, Colombia; lfvalladales@utp.edu.co; 2 Grupo de Investigación Biomedicina, Facultad de Medicina, Fundación Universitaria Autónoma de las Américas, Pereira 660001, Colombia; 3 Semillero de Investigación en Farmacología Geriátrica, Grupo de Investigación Biomedicina, Facultad de Medicina, Fundación Universitaria Autónoma de las Américas, Pereira 660001, Colombia; lian.soto@uam.edu.co (L.M.S.-R.); luis.navarrete@uam.edu.co (L.F.N.-S.);; 4 Grupo Operador Clínico, Área de Salud, Cali 760045, Colombia; asesormodelogr@ospedale.com.co

**Keywords:** amphotericin B, antifungal agents, mortality, mycoses, pharmacoepidemiology, Colombia

## Abstract

**Background/Objectives**: Amphotericin B is indicated in deep systemic fungal infections. The aim was to determine the sociodemographic, clinical and pharmacological variables of a group of Colombian patients treated with amphotericin B and factors associated with mo rtality. **Methods**: A longitudinal observational retrospective study on the use of amphotericin B in Colombia was conducted between January 2015 and December 2022. The multivariate analysis sought to identify variables related to mortality. **Results**: A total of 310 patients were identified, with a median age of 44.0 years, and 71.0% were women. Conventional amphotericin B was the most used (74.8%). The main uses were cryptococcosis (38.7%), histoplasmosis (31.9%) and candidiasis (29.4%). More than a third of patients died during hospitalization (40.3%). An increase in the Charlson Comorbidity Index score (HR: 1.13; 95% CI: 1.05–1.22) and in the qSOFA score (HR: 1.34; 95% CI: 1.04–1.73), coinfection by *Mycobacterium tuberculosis* (HR: 2.09; 95% CI: 1.32–3.31) and the requirement of vasopressors (HR: 4.20; 95% CI: 2.16–8.15) or invasive mechanical ventilation (HR: 2.73; 95% CI: 1.40–5.33) increased the probability of in-hospital death. In contrast, those who received systemic corticosteroids (HR: 0.43; 95% CI: 0.26–0.70) had a lower risk. Conventional amphotericin B is the most used drug mainly treating *Cryptococcus neoformans* infections. **Conclusions**: The use of amphotericin B was consistent with clinical practice guideline recommendations. In-hospital mortality was common, and factors such as increased comorbidities, higher qSOFA scores, coinfection with *Mycobacterium tuberculosis* and invasive procedures like mechanical ventilation were linked to increased mortality.

## 1. Introduction

Invasive fungal infections affect more than 6.5 million people each year and lead to the death of more than 3.7 million people worldwide [1]. Thus, the World Health Organization (WHO) considers pathogens and fungal infections to be an increasing public health problem [2]. The morbidity and mortality caused by invasive fungal infections is continuously increasing due to several factors [2,3,4]. There is a considerable increase in the number of patients with disease-induced or drug-induced immunodeficiencies (the population group most vulnerable to these infections). Further, there has been an increase in drug-resistant fungal pathogens, and the therapeutic arsenal available to treat them is limited; there are only three main classes or families of antifungal drugs: azoles, echinocandins and polyenes [2,3,4].

Among the polyenes, conventional amphotericin B (amphotericin B deoxycholate) has been used in clinical practice on a regular basis since its authorization and marketing in 1959 [5]. This is because, after more than 60 years of clinical use, its therapeutic properties—such as having the largest known spectrum of antifungal action—remain intact, and acquired resistance to the drug is rare [6]. However, this drug is poorly tolerated compared to other systemic antifungals, and individuals report greater adverse drug reactions, especially hypokalemia and nephrotoxicity [7]. To reduce the intrinsic toxicity of conventional amphotericin B, several lipid formulations of amphotericin B were developed during the 1990s, such as liposomal amphotericin B, amphotericin B colloidal dispersion and the amphotericin B lipid complex, which often have a better safety profile and the same efficacy [8,9]. However, in some countries, the high cost of these lipid-based formulations limits their routine use [9,10].

The Colombian Health System offers universal coverage to the entire population through two regimes, one contributory or paid by workers and employers and the other subsidized by the state, which has a benefit plan that covers several pharmaceutical forms of amphotericin B. However, recent information available in the country on the use of systemic antifungals is limited [11]. A recent study evaluated the use patterns and indications of antifungals dispensed in outpatients, using a pharmacy manager’s database. But because amphotericin B is a drug administered intravenously, it was not included in the study [11]. Internationally, studies involving the use of amphotericin B with real-world evidence are scarce [12,13], and there are no data available in Colombia. Thus, in a low–middle-income country like ours, the patterns of use, indications, doses, complications and mortality in patients treated with amphotericin B are unknown. Therefore, the objective of this study was to determine the sociodemographic, clinical and pharmacological variables of a group of Colombian patients treated with amphotericin B and the factors associated with mortality.

## 2. Results

### 2.1. Sociodemographic

**Figure 1:**
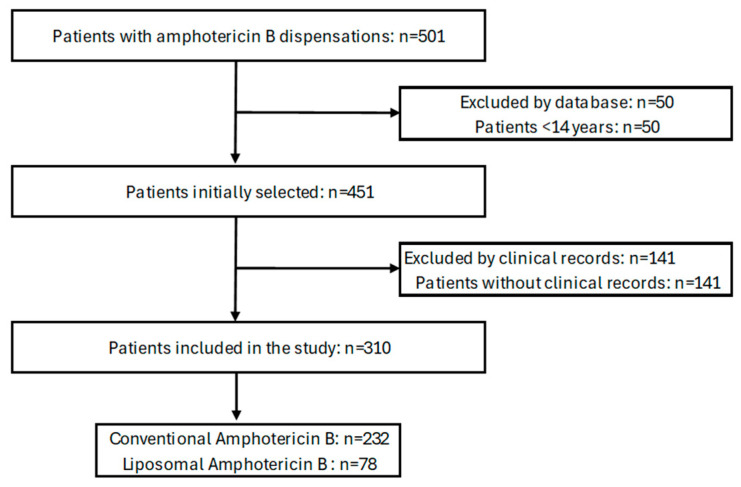
Study flow diagram.

This study included 310 patients treated with amphotericin B, coming from 60 different cities (Figure 1). A total of 71.0% were men, and the median age was 44.0 years (interquartile range: 34.8–58.0 years). A total of 37.7% (*n* = 117) were between 14 and 39 years old, 47.1% (*n* = 146) were between 40 and 64 years old, and 15.2% (*n* = 47) were 65 or older. Most were in the Pacific region, had primary or secondary education (*n* = 147; 47.4%), carried out household activities (*n* = 32; 10.3%) and were affiliated with the subsidized health insurance scheme (see Table 1).

### 2.2. Clinics

The median CCI score was six points, and the majority had diagnoses of HIV infection, followed by arterial hypertension and some type of neoplasia (Table 1). The baseline vital signs of the patients are shown in Table 1. The median duration of hospitalization was 26.0 days (interquartile range: 15.0–43.0). A total of 58.7% (*n* = 182) required ICU management, 83.5% (*n* = 259) required supplemental oxygen, and 46.1% (*n* = 143) required invasive mechanical ventilation, with a median of 4.0 days (interquartile range:1.0–11.0) of intubation time, and 7.4% (*n* = 23) underwent tracheostomy. A total of 19.4% (*n* = 60) had some surgical intervention. The main surgeries performed were laparotomy (*n* = 10; 3.2%), craniotomy (*n* = 7; 2.3%), biopsy (*n* = 6; 1.9%), pulmonary decortication (*n* = 4; 1.3%) and collection drainage (*n* = 4; 1.3%). A total of 21.6% (*n* = 67) had concomitant infections by *Mycobacterium tuberculosis*, 7.4% (*n* = 23) by *Pneumocystis jirovecii*, 6.5% (*n* = 20) by herpes viruses, 5.8% (*n* = 18) by cytomegalovirus, 5.2% (*n* = 16) by SARS-CoV-2 and 2.3% (*n* = 7) by *Toxoplasma gondii*. The most common invasive fungal infections in patients with SARS-CoV-2 were histoplasmosis (*n* = 5), candidiasis (*n* = 4) and aspergillosis (*n* = 3).

### 2.3. Pharmacological Treatment

A total of 43.9% (*n* = 136) of the patients received some systemic antifungals before starting with amphotericin B. These antifungals were administered mainly by the parenteral route (51.5%; *n* = 70/136) followed by the oral route (48.5%; *n* = 60). The median from admission to the start of amphotericin B was 8.0 days (interquartile range: 4.0–17.8). Conventional amphotericin B was the most used (74.8%), followed by liposomal amphotericin B. The dose and treatment time can be seen in Table 2. The median time of amphotericin B infusion was 4 h (interquartile range: 4.0–4.0).

In 91.6% (*n* = 284) of the patients, it was used for the management of infections, while in 7.1% (*n* = 22), it was used as prophylaxis, and in 1.3% (*n* = 4) of the cases, it was not possible to identify indications in the clinical records. The most frequent uses were for the management of cryptococcosis (*n* = 120; 38.7%), histoplasmosis (*n* = 99; 31.9%) and candidiasis (*n* = 91; 29.4%). A total of 20.0% (*n* = 62) of the patients had two concomitant systemic fungal infections. Most presented histoplasmosis with candidiasis (*n* = 20; 6.5%), followed by cryptococcosis with candidiasis (*n* = 18; 5.8%) and histoplasmosis with cryptococcosis (*n* = 16; 5.2%). The median time of treatment was 7.0 days (interquartile range: 3.0–14.0) and was higher in the group with a diagnosis of cryptococcosis (9.0 days; interquartile range: 4.0–16.0). Table 2 shows the male/female ratio, age, dose, duration of treatment and indications by type of amphotericin B. A total of 11.9% (*n* = 37) of the patients had a change in the type of amphotericin B, especially from conventional to liposomal amphotericin B (*n* = 33; 10.6%).

The comedications most frequently received by patients during their hospital stay were other systemic anti-infectives (*n* = 305; 98.4%), followed by antiulcers (*n* = 297; 95.8%), analgesics and anti-inflammatories (*n* = 291; 93.9%), systemic glucocorticoids (*n* = 213; 68.7%), anticoagulants (*n* = 214; 69.0%), benzodiazepines and analogs (*n* = 191; 61.6%), antihypertensives and diuretics (*n* = 185, 59.7%), vasopressors and inotropics (*n* = 131, 42.3%), antihistamines (*n* = 97, 31.3%) and antipsychotics (*n* = 83, 26.8%). Regarding anti-infectives, the majority received antibiotics (*n* = 300; 96.8%), other antifungals (*n* = 239; 77.1%), especially imidazoles (*n* = 225; 72.6%), antivirals (*n* = 96, 31.0%), antiparasitics (*n* = 93, 30.0%) and antiretrovirals (*n* = 52, 16.8%). The most used imidazoles were fluconazole (*n* = 193; 62.3%), followed by itraconazole (*n* = 37; 11.9%), posaconazole (*n* = 12; 3.9%) and voriconazole (*n* = 10; 3.2%).

### 2.4. Laboratory

The hemogram, liver and kidney function, and baseline electrolytes are shown in Table 1. Laboratory values were compared before starting amphotericin B and at the end of the treatment scheme, and significant changes were found in hemoglobin (median: 9.60 mg/dL vs. 8.90 mg/dL; *p* = 0.001), sodium (135.0 mEq/L vs. 137.0 mEq/L; *p* = 0.008), creatinine (0.78 mg/dL vs. 0.98 mg/dL; *p* < 0.001) and glomerular filtration rate (107.7 mL/min vs. 93.5 mL/min; *p* < 0.001) but not for potassium (3.90 mEq/L vs. 3.74 mEq/L; *p* = 0.147).

### 2.5. Complications

The median qSOFA score was 1 point (interquartile range: 0–1), and 12.3% (*n* = 38) had a qSOFA score of ≥2 points at admission. A total of 34.2% (*n* = 106) of the patients were diagnosed with sepsis during hospitalization, 8.7% (*n* = 27) experienced septic shock, and 40.3% (*n* = 125) died. The median between admission and death was 18.0 days (interquartile range: 9.0–30.0).

### 2.6. Multivariate Analysis

Our Cox proportional hazards regression analysis showed that the increase in the CCI score, the increase in the qSOFA score, coinfection by *Mycobacterium tuberculosis*, and the need for vasopressors or invasive mechanical ventilation increased the probability of in-hospital death; on the other hand, those who received systemic glucocorticoids had a lower risk (Table 3).

## 3. Discussion

This study allowed us to identify the uses, complications and mortality of patients who were managed with amphotericin B, as well as the variables that may be related to mortality, as evidence of the use of drugs in the real world. Amphotericin B was used primarily for the treatment of cryptococcosis. Complications such as sepsis and in-hospital mortality occurred in more than a third of patients. Knowledge together with real-world evidence of the specific use practice of amphotericin B can be useful for infectious disease physicians and other physicians who treat patients with fungal infections, since the evidence provides them with elements that expand their knowledge about the most frequently identified microorganisms and the doses tolerated by patients in the country. These findings may be useful for health care, academic and scientific personnel in making decisions regarding the risks faced by their patients and contribute to strengthening the practices of rational use of antimicrobials among physicians.

The median age of the patients was like that found in Brazil (43.0 years) [14] and lower than that found in other studies (44.0 vs. 52.0–68.4 years) [13,15,16,17], with a predominance of men as identified in different studies (71.0% vs. 54.3–65.0%) [12,13,14,15,16,17]. The predominance in men may be due to greater environmental exposure to fungi due to work activities [18]. In addition, the development of a better immune response in women makes them more resistant to fungal infections compared to men [18]. Serious fungal infections occur because of other health problems, including HIV infection and cancer [1], which were prevalent in this study as well as in other studies [13,16]. Hereditary, acquired or iatrogenic immunodeficiency predispose patients to opportunistic fungal infections [3]. This is why the use of antifungals is typical in this group of patients [2,3,4]. In this way, the use of other antifungal drugs before starting treatment with amphotericin B is common, as evidenced in two reports made in Japan (47.6% and 64.0%) [15,16].

Conventional amphotericin B was the most widely used pharmaceutical form in this patient group, which is consistent with that reported in Australia (66.1%) [12] and Brazil (48.6–54.7%) [13,14]. This predominance is probably due to the significantly lower cost of this presentation compared to lipid forms such as liposomal or lipid complexes (USD 12.4 vs. USD 2143–2213) [10]. This antifungal was used mainly for the management of cryptococcosis cases, which is consistent with that described by Falci et al. in a study with real-world evidence conducted in Brazil (45.1%) [13]. In Japan, it was used mainly for the management of cases of aspergillosis (22.0%–25.0%) [16,17]. The pattern of use of antifungals may vary according to the global epidemiological differences in invasive mycoses [1]. The uses and doses used are in line with what was approved by the regulatory agencies [19,20]. The use of amphotericin B (mainly conventional) is associated with renal toxicity, hypokalemia and anemia [21]. The current study showed a reduction in glomerular filtration rate and hemoglobin. However, because this was a retrospective study, the causal relationship between the drug and these adverse events could not be determined.

In-hospital mortality was 40.3%, which was very similar to that found in China (41.2%) [22] and Japan (42.6%) [16] and lower than that found in Brazil (54.3–54.6%) [13,23]. Some variables were found that were related to this outcome. Patients who had a higher CCI score also had a higher risk of dying, which is consistent with other reports [20,24]. Higher scores indicate a higher risk of mortality but also more serious comorbid conditions [25]. The CCI score was developed to be used in different populations as a prognostic measure to predict mortality [25]. Likewise, the higher the qSOFA score, the higher the probability of dying, which is in line with findings described by Xie et al. in China [22]. The qSOFA score has a good positive predictive value for identifying patients with sepsis outside the ICU and predicting in-hospital mortality [26]. In addition, patients who required management with vasopressors or inotropes and with mechanical ventilation also presented a higher risk, consistent with what was found in the literature [13,23,24]. Acid–base disorders, hypoperfusion and oxygenation disorders are managed with vasopressors, inotropics and medical devices; however, despite this, these clinical conditions are associated with high mortality [27].

It was found that the use of glucocorticoids was a predictor of survival. This finding is consistent with some systematic reviews and meta-analyses of randomized clinical trials of patients with sepsis where it was shown that glucocorticoids reduce mortality [28,29]. These drugs inhibit the systemic inflammatory response and tissue damage, improve tolerance to bacterial endotoxins and restore microcirculatory hemodynamics [29]. They also downregulate uncontrolled pro-inflammatory responses, inhibit excessive consumption of immune factors and cells and maintain the body’s innate immune capacity [29]. However, in another systematic review and meta-analysis that involved analytical and observational studies, it was shown that the use of glucocorticoids in patients with aspergillosis, invasive candidiasis and mucormycosis increased the risk of dying [30].

Certain limitations inherent to observational studies should be considered when interpreting the results of the current study. This study did not select the subjects through sampling but rather included all patients treated with amphotericin B (Figure 1). A selection bias may be present because the patients come from four clinics, and these are not representative of the entire Colombian population, so the data may not be extrapolated to other healthcare centers. This study also has a retrospective longitudinal design and the information was collected from clinical records, so there are possibly data collection biases. Thus, for the variables of some patients, complete information was not available, especially for the reporting of some of the laboratory analyzes. The sensitivity and resistance pattern of the microorganisms was not identified. Furthermore, the clinical registries did not have analyses of causal relationships between adverse events and the use of amphotericin B. Therefore, it was not possible to describe the adverse drug reactions associated with this antifungal. The causes that led to the changes in antifungal agents during the patients’ hospitalization could not be determined either. Finally, potential confounding biases that may have occurred in this study were controlled through multivariate analyzes (Cox regression). However, this study provides real-world evidence from patients as young as 14 years of age that may be useful to physicians treating systemic fungal infections.

## 4. Materials and Methods

### 4.1. Study Design and Patients

A longitudinal observational retrospective study was carried out on the sociodemographic, clinical, paraclinical and pharmacological characteristics of the patients who required treatment with amphotericin B and the factors related to in-hospital mortality. The patients were identified from the dispensing of the different pharmaceutical forms of amphotericin B carried out by the company Audifarma SA in four clinics of the Ospedale Group network located in the cities of Bogotá, Cali, Pereira and Popayán.

The records of all patients aged 14 and older of any sex and city of residence dated between 1 January 2015 and 31 December 2022 were selected. Patients with incomplete medical records were excluded. Regarding the selected cases, the electronic medical records that were consigned during the observation and follow-up period (until hospital discharge or death) were reviewed (Figure 1).

### 4.2. Variables

From the information obtained, a database was designed that allowed the following groups of variables to be collected:

1. Sociodemographic data: sex, age, occupation, education, affiliation regime (contributory or subsidized) and place of origin. The place of origin was categorized by departments according to the regions of Colombia, considering the classification of the National Administrative Department of Statistics (DANE) of Colombia, as follows: Caribbean, Central, Bogotá–Cundinamarca, Pacific and Eastern-Orinoquia–Amazon.
2. Clinics:
  (a) Physiological variables: systolic blood pressure, diastolic blood pressure, heart rate, respiratory rate, oxygen saturation and state of consciousness (Glasgow) at the time of initial care.
  (b) Anthropometric measurements: weight, height and body mass index.
  (c) Diagnosis: The type of systemic fungal infection present in each patient was determined (*Cryptococcus neoformans, Histoplasma capsulatum, Candida albicans* and other *Candidas, Aspergillus* spp., *Mucor* spp., etc.), as well as other coinfections (*Mycobacterium tuberculosis, Pneumocystis jirovecii*, cytomegalovirus, and *Toxoplasma gondii*, among others). The diagnoses were taken from the patients’ clinical records, which were based on the clinical criteria of the internist or infectious disease physician, as well as laboratory studies (microscopic studies, cultures, molecular studies, etc.). *Candida lusitaniae* and *Aspergillus terreus* are not sensitive to amphotericin B.
  (d) Comorbidities: solid or hematological neoplasms, human immunodeficiency virus (HIV), rheumatological diseases (rheumatoid arthritis, systemic lupus erythematosus, vasculitis, and others), chronic kidney disease, liver cirrhosis, high blood pressure, and diabetes mellitus, among others. An age-adjusted Charlson Comorbidity Index (CCI) score was calculated.
  (e) Complications: sepsis, septic shock and in-hospital mortality were identified. A Quick Sequential Organ Failure Assessment (qSOFA) score was calculated.
3. Laboratory: complete blood count, total bilirubin, direct bilirubin, aspartate aminotransferase, alanine aminotransferase, lactate dehydrogenase, electrolytes (sodium and potassium), urea nitrogen, and creatinine, at the time of initial care, before starting amphotericin B therapy and at the end of therapy. The glomerular filtration rate (GFR) was calculated using the Chronic Kidney Disease Epidemiology Collaboration (CKD-EPI) 2021 equation.
4. Therapeutics/Management:
  (a) Place of care: hospitalization in general wards and intensive care units (ICUs).
  (b) Use of supplemental oxygen: oxygen requirement, mechanical ventilation and need for tracheostomy.
  (c) Amphotericin B: type (conventional, lipid complex, liposomal or colloidal dispersion), dose used (mg per day), defined daily dose (DDD), duration of therapy, time of infusion, changes in the type of amphotericin B, indications (aspergillosis, candidiasis, cryptococcosis, and histoplasmosis, among others), monotherapy vs. combined antifungal therapy (azoles and echinocandins, among others), and prophylactic or therapeutic use.
  (d) Comedications: vasopressors and inotropics, antihypertensives and diuretics, normoglycemic agents, antiulcers, other systemic antimicrobials (antibiotics, antivirals, etc.), anticoagulants, analgesics and anti-inflammatories, benzodiazepines, systemic corticosteroids, antipsychotics, and antihistamines, among others.

### 4.3. Ethics Statement

The study protocol was endorsed by the Bioethics Committee of the Technological University of Pereira in the category of “research without risk” (approval code: 48-080822). The principles of confidentiality of information established by the Declaration of Helsinki were respected. The laws in Colombia (Resolution 8430 of 1993 of the Ministry of Health) exempt researchers from needing to obtain informed consent for risk-free research as the information is obtained from electronic records.

### 4.4. Data Analysis

The data were analyzed with the statistical package SPSS Statistics, version 26.0 for Windows (IBM, Armonk, New York, NY, USA). A descriptive analysis was performed with frequencies and proportions for the qualitative variables and measures of central tendency and dispersion for the quantitative variables through medians and interquartile ranges. A comparison of the quantitative variables was performed using the Mann—Whitney U test and X 2 or Fisher’s exact test for categorical variables. Cox proportional hazards regression was performed, taking the duration of hospitalization in days as the follow-up time and whose dependent variable was in-hospital mortality (yes/no). The covariates included in the models were those that showed association in the bivariate analyses, as well as those with sufficient plausibility or reported association. A level of statistical significance was established at *p* < 0.05.

## 5. Conclusions

With these findings, we can conclude that in this group of hospitalized patients, conventional amphotericin B was the most widely used pharmaceutical form, mainly for the management of *Cryptococcus neoformans* infections. The use of amphotericin B was consistent with clinical practice guideline recommendations. Complications and in-hospital mortality were common. The number of comorbidities, a higher qSOFA score, concomitant tuberculosis, and the use of vasopressors or invasive mechanical ventilation were associated with a greater probability of dying during hospitalization. However, the use of systemic corticosteroids was associated with a better prognosis.

## Figures and Tables

**Table 1:** Sociodemographic, clinical and paraclinical variables of a group of patients treated with amphotericin B in Colombia.

Variables	Total	
	*n* = 310	%
Sociodemographic		
Male	220	71.0
Age, median (IQR)	44.0 (34.8–58.0)	
Region of origin		
Pacific	173	55.8
Central	112	36.1
Bogotá–Cundinamarca	22	7.1
Eastern-Orinoquia-Amazon	3	1.0
Affiliation regime	-	-
Subsidized	166	53.5
Contributory	144	46.5
Comorbidities		
Charlson index, median (IQR)	6 (2–7)	
0 points	40	12.9
1–2 points	50	16.1
≥3 points	220	71.0
Human immunodeficiency virus infection	173	55.8
Arterial hypertension	82	26.5
Hematological or solid neoplasms	63	20.3
Chronic kidney disease	58	18.7
Diabetes mellitus	47	15.2
Vital signs (on admission), median (IQR)		
Systolic blood pressure (mmHg)	115.0 (100.0–125.0)	
Diastolic blood pressure (mmHg)	70.0 (60.0–78.0)	
Heart rate (bpm)	85.0 (78.0–98.3)	
Respiratory rate (rpm)	20.0 (18.0–21.0)	
Temperature (°C)	36.5 (36.0–36.9)	
Oxygen saturation (%)	95.0 (94.0–97.0)	
State of consciousness (Glasgow)	15.0 (15.0–15.0)	
Anthropometric measurements, median (IQR)		
Weight (kg)	57.0 (50.0–65.0)	
Height (m)	1.7 (1.6–1.7)	
Body mass index (kg/m^2^)	21.5 (18.8–24.2)	
Laboratory studies, median (IQR)		
Hemogram		
Hemoglobin (g/dL)	11.0 (8.8–13.1)	
Hematocrit (%)	32.9 (27.2–39.0)	
Leukocytes (/mm³)	7250.0 (4040.0–11600)	
Neutrophils (/mm³)	4545.0 (2235.0–8392.5)	
Lymphocytes (/mm³)	840.0 (475.0–1500.0)	
Platelets (/mm³)	204,000.0 (112,350.0–293,000.0)	
Renal function		
Creatinine (mg/dL)	0.82 (0.66–1.09)	
Glomerular filtration rate (mL/min) ^a^	104.8 (74.8–119.3)	
Urea nitrogen (mg/dL)	15.3 (11.0–21.9)	
Liver function		
Total bilirubin (mg/dL)	0.59 (0.38–0.94)	
Direct bilirubin (mg/dL)	0.33 (0.21–0.59)	
Alanine aminotransferase (U/L)	33.0 (18.0–56.9)	
Aspartate aminotransferase (U/L)	36.0 (22.0–75.0)	
Lactic acid dehydrogenase (U/L)	308.0 (190.8–694.8)	
Electrolytes		
Sodium (mEq/L)	135.0 (131.0–139.0)	
Potassium (mEq/L)	4.0 (3.6–4.4)	
Isolated microorganisms		
*Cryptococcus neoformans*	106	34.2
*Histoplasma capsulatum*	99	31.9
*Candida albicans*	63	20.3
Other *Candida*	28	9.0
*Aspergillus* spp.	17	5.5
*Cryptococcus* spp.	14	4.5

IQR: interquartile range; bpm: beats per minute; rpm: breaths per minute. ^a^ The glomerular filtration rate was calculated using the Chronic Kidney Disease Epidemiology Collaboration (CKD-EPI) 2021 equation.

**Table 2:** Comparison of sex, age, dose, days of treatment and indications, between conventional and liposomal amphotericin B, in Colombia.

Variables	Conventional Amphotericin B		Liposomal Amphotericin B	
	*n* = 232	%	*n* = 78	%
Sex				
Men	166	71.6	54	69.2
Women	66	28.4	24	30.8
Age				
Mean ± SD	47.9 ± 16.5		42.6 ± 16.2	
Median (IQR)	46.0 (37.0–59.0)		40.5 (29.0–51.3)	
Prescribed dose				
Mean ± SD	44.0 ± 10.9		191.3 ± 47.6	
Median (IQR)	50.0 (35.0–50.0)		197.5 (150.0–200.0)	
Mode	50.0		200.0	
nDDD ^a^	1.25			
Treatment days				
Mean ± SD	8.5 ± 6.8		9.1 ± 7.3	
Median (IQR)	6.0 (3.0–14.0)		8.0 (2.0–14.0)	
Indications				
Treatment	214	92.2	74	94.9
Cryptococcosis	92	39.7	28	35.9
Histoplasmosis	69	29.7	30	38.5
Candidiasis	69	29.7	22	28.2
Aspergillosis	15	6.5	3	3.8
Mucormycosis	9	3.9	1	1.3
Blastomycosis	1	0.4	1	1.3
Sporotrichosis	1	0.4	1	1.3
Coccidioidomycosis	0	0.0	1	1.3
Leishmaniasis	1	0.4	0	0.0
Microsporidiasis	1	0.4	0	0.0
Trichosporonosis	1	0.4	0	0.0
No data	3	1.3	1	1.3
Prophylaxis	18	7.8	4	5.1

SD: standard deviation; IQR: interquartile range; ^a^ Proportion between the daily dose received and the defined daily dose.

**Table 3:** Cox proportional hazards regression of the variables related to in-hospital mortality in a group of patients who were treated with amphotericin B, Colombia.

Variables	Sig.	HR	95% CI	
			Lower	Upper
Man (yes/no)	0.774	1.062	0.704	1.603
Age (continuous)	0.231	0.990	0.974	1.006
Central-region origin (yes/no)	0.249	0.766	0.487	1.205
Arterial hypertension (yes/no)	0.880	1.037	0.644	1.670
Charlson Comorbidity Index (continuous)	<0.001	1.136	1.058	1.220
qSOFA (continuous)	0.020	1.348	1.049	1.731
Glomerular filtration rate on admission (continuous)	0.599	1.002	0.995	1.009
*Mycobacterium tuberculosis* infection (yes/no)	0.002	2.095	1.326	3.311
Cryptococcosis (yes/no)	0.804	1.051	0.709	1.558
Conventional amphotericin B (yes/no)	0.200	0.767	0.512	1.150
Fluconazole (yes/no)	0.422	0.852	0.577	1.259
Systemic corticosteroids (yes/no)	0.001	0.434	0.267	0.706
Vasopressors and/or inotropes (yes/no)	<0.001	4.205	2.167	8.157
Invasive mechanical ventilation (yes/no)	0.003	2.734	1.401	5.332

Sig.: statistical significance; HR: hazard ratio; CI: confidence interval; qSOFA: Quick Sequential Organ Failure Assessment.

## Data Availability

protocolos.io. https://www.protocols.io/private/70EBB40C6DCA11EEA7690A58A9FEAC02.
